# Late Femoral Component Revision for Femoral Canal Perforation

**DOI:** 10.7759/cureus.20996

**Published:** 2022-01-06

**Authors:** Gregory K Deirmengian, Jeffrey Lynch, Stephanie Kwan, Brian Fliegel

**Affiliations:** 1 Orthopaedic Surgery, Rothman Orthopaedic Institute, Philadelphia, USA; 2 Orthopaedic Surgery, Rowan University School of Osteopathic Medicine, Stratford, USA

**Keywords:** total joint replacement, total joint arthroplasty, femoral canal perforation, total hip revision, failed total hip, total hip arthroplasty: tha

## Abstract

Femoral perforation during total hip arthroplasty is a rare complication. Most of the existing literature regarding the complication involves acutely recognized perforations. We report a case of femoral component revision for a symptomatic femoral perforation 12 years after a primary cementless total hip arthroplasty. The revision allowed for intramedullary component positioning, restoration of femoral length and offset, pain relief, and functional improvement. While management of this complication is debatable, we recommend revision in order to avoid future complications and optimize patient outcomes.

## Introduction

Femoral perforation is a rare complication of total hip arthroplasty with an incidence that has not been delineated in the literature [1-3]. Most of the existing literature on the topic focuses on the prevention and management of complications in the intraoperative and immediate postoperative periods [1-7]. Patient risk factors for femoral perforation include previous surgery, hip dysplasia, and osteoporosis [2]. Surgical factors predisposing to femoral perforation include poor exposure and the use of a long-stemmed implant [3]. When recognized in the intraoperative setting, treatment involves removal of the existing component and bypassing the cortical defect with a longer stemmed component [1-5]. Alternatively, a standard component may be placed in the appropriate position and the cortical defect can be reinforced with a strut graft or plate and screw device [7].

When femoral perforation is first recognized in the postoperative setting, management options include immediate revision and nonoperative treatment with a period of protected weight bearing [1,6]. While satisfactory long-term outcome with nonoperative management has been reported [3], concern has been raised regarding the long-term risk of periprosthetic fracture [5]. We report the revision of a cementless femoral component for femoral perforation 12 years after the index procedure.

## Case presentation

The patent is a 37-year-old male who, 12 years prior to presentation, underwent a left primary total hip arthroplasty for post-traumatic avascular necrosis of the femoral head. The initial operative report noted a “great deal of difficulty obtaining access to the intramedullary canal due to previous trauma and stenosis.” In addition, the surgeon noted “a great deal of difficulty getting the (smallest-sized) broach down the canal due to sclerotic bone.” The patient had an uneventful postoperative course, though he had residual postoperative pain that never improved and was unable to ambulate without a cane. The patient did not recall any discussions with his index surgeon regarding radiographic abnormalities.

**Figure 1:**
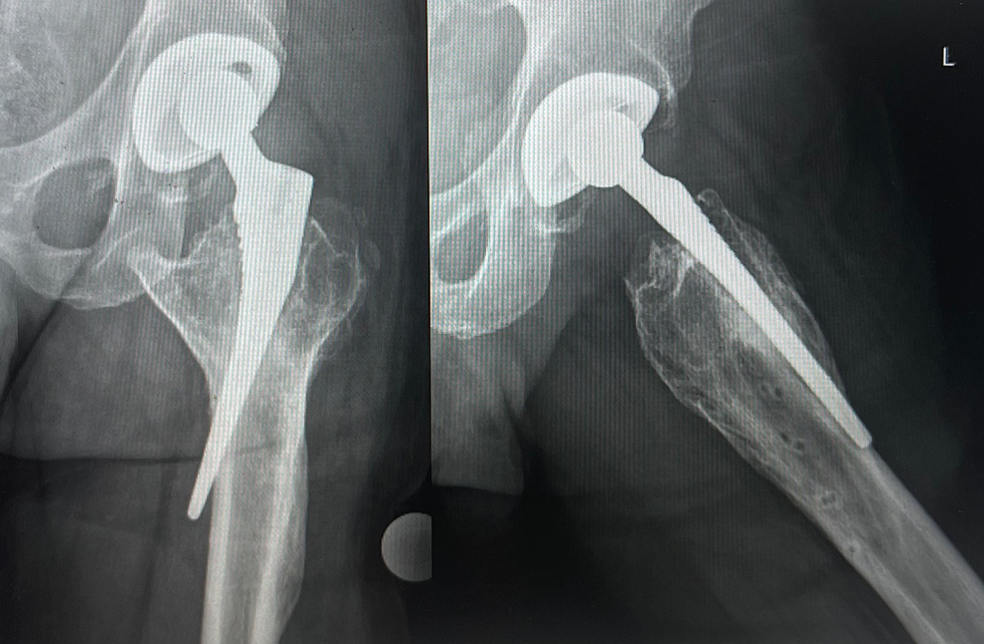
Anteroposterior and lateral hip radiographic images demonstrating femoral component malpositioning and perforation

**Figure 2:**
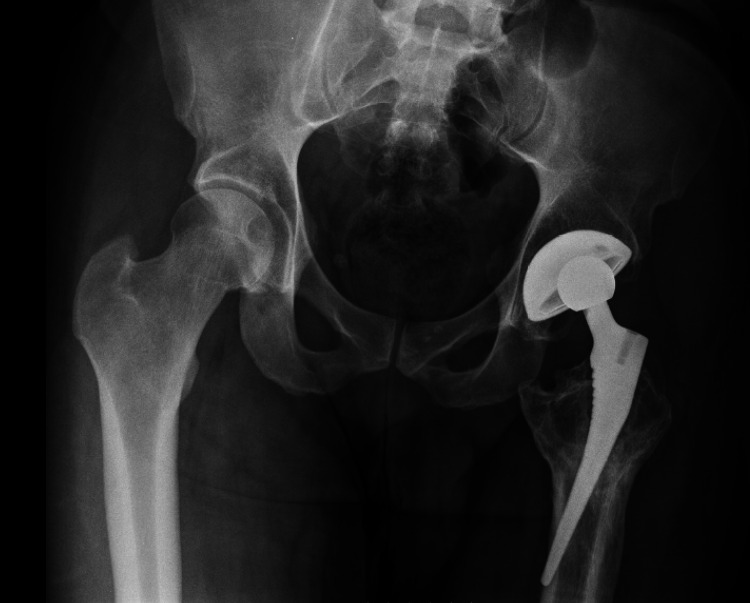
Anteroposterior pelvic radiographic image demonstrating pelvic tilt

The patient presented for a second opinion 12 years after the index procedure with complaints of persistent thigh pain, subjective lower extremity weakness, and reliance on a cane since his initial surgery. The patient did not follow up with his surgeon after his initial early postoperative visits and, after many years of living with his symptoms, was convinced by his family to seek another opinion after his symptoms were exacerbated by a fall. Physical examination revealed pelvic tilt, a marked limp with gait, pain with Stinchfield testing, and 20 mm of left lower extremity lengthening. Hip radiographs (Figure 1) revealed femoral component malpositioning, involving anteromedial femoral perforation, as well as signs of a partial fibrous ingrowth. In addition, a pelvic radiograph revealed relative left leg lengthening and substantial loss of offset (Figure 2). Lastly, radiographs revealed mild acetabular polyethylene liner wear. The patient was found to have a C-reactive protein level and an erythrocyte sedimentation rate within normal limits. Considering the patient's symptoms, the findings on the clinical history and physical examination, and the radiographic and laboratory findings, the decision was made to proceed with hip revision surgery.

**Figure 3:**
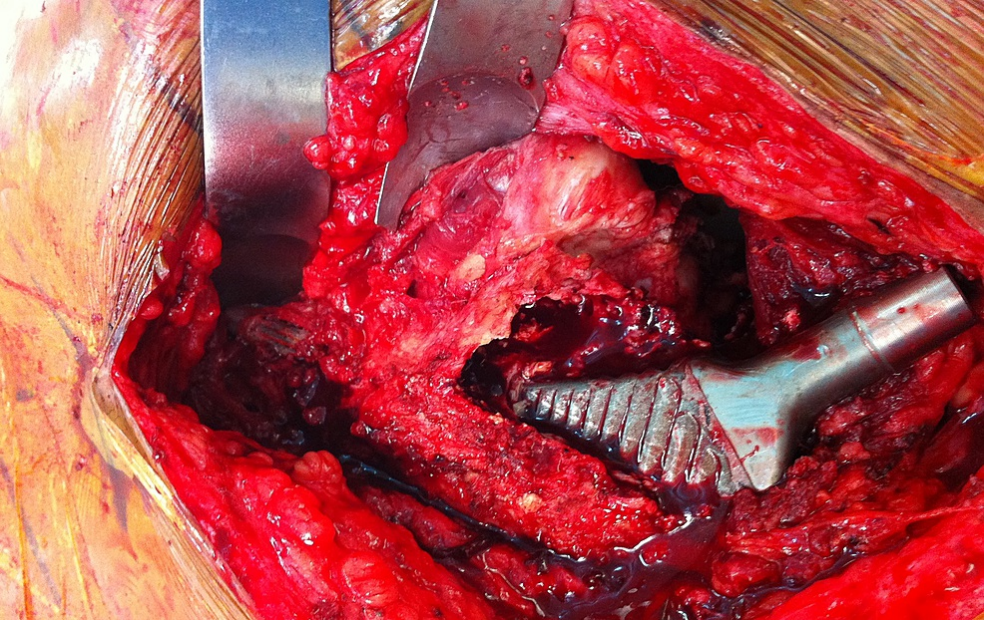
Intraoperative clinical image demonstrating the ingrown femoral component

**Figure 4:**
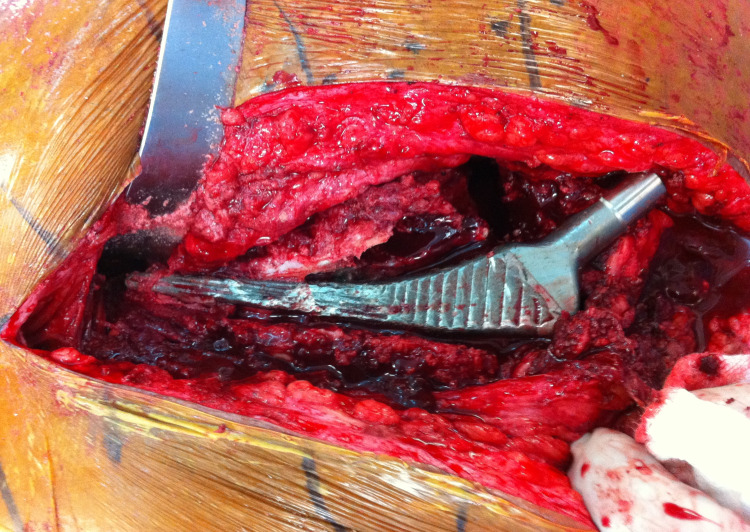
Intraoperative clinical image after exposure of the entire anterior aspect of the femoral component

**Figure 5:**
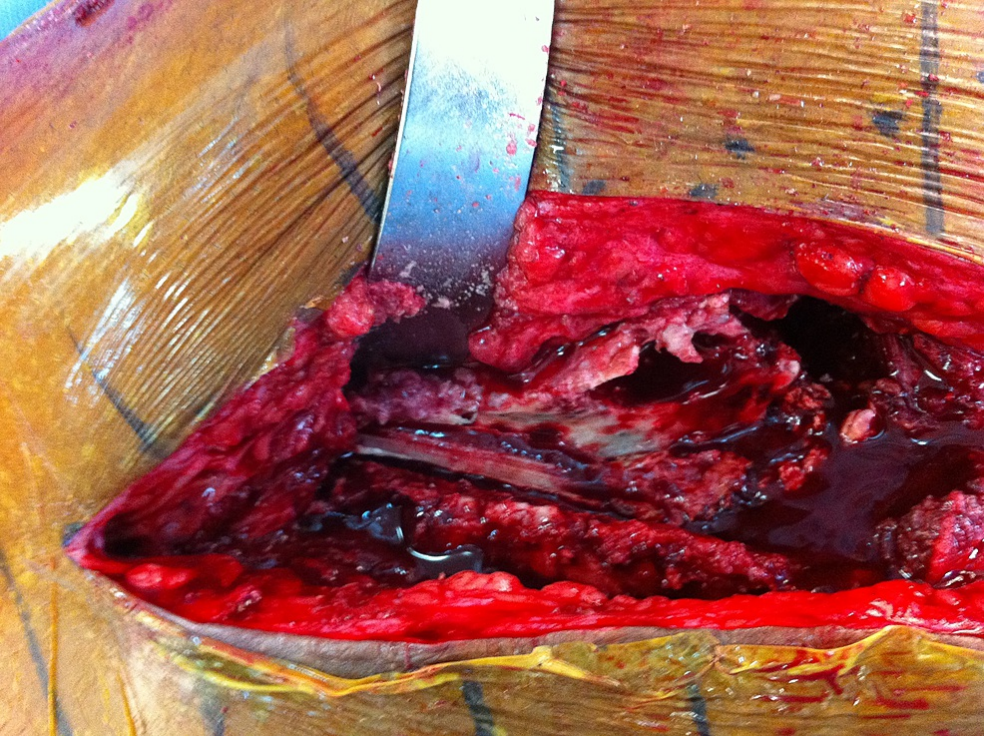
Intraoperative clinical image after exposure of the entire anterior aspect of the femur after component removal

From a surgical standpoint, the hip was exposed through a modified-Hardinge approach with the patient in the supine position. After dislocating the hip, removing the femoral head, and exposing the acetabulum, the acetabular liner was removed. After confirming rigid fixation of the acetabular component, the existing locking ring was exchanged and a new polyethylene liner was secured to the acetabular component using the locking mechanism. Turning attention to the femoral component, the vastus lateralis was split in order to expose the femoral component and identify the femoral perforation (Figure 3). The anterior aspect of the femoral component was found to have a stable fibrous ingrowth to the proximal femur. At this point, the anterior proximal femoral bone was removed, exposing the entire anterior aspect of the femoral component (Figure 4). The posterior aspect of the component was found to have solid bony ingrowth. A burr was used to carefully detach the posterior aspect of the component from the surrounding bone. Subsequent removal of the component by hand revealed the remaining aspect of the femoral cortex (Figure 5).

**Figure 6:**
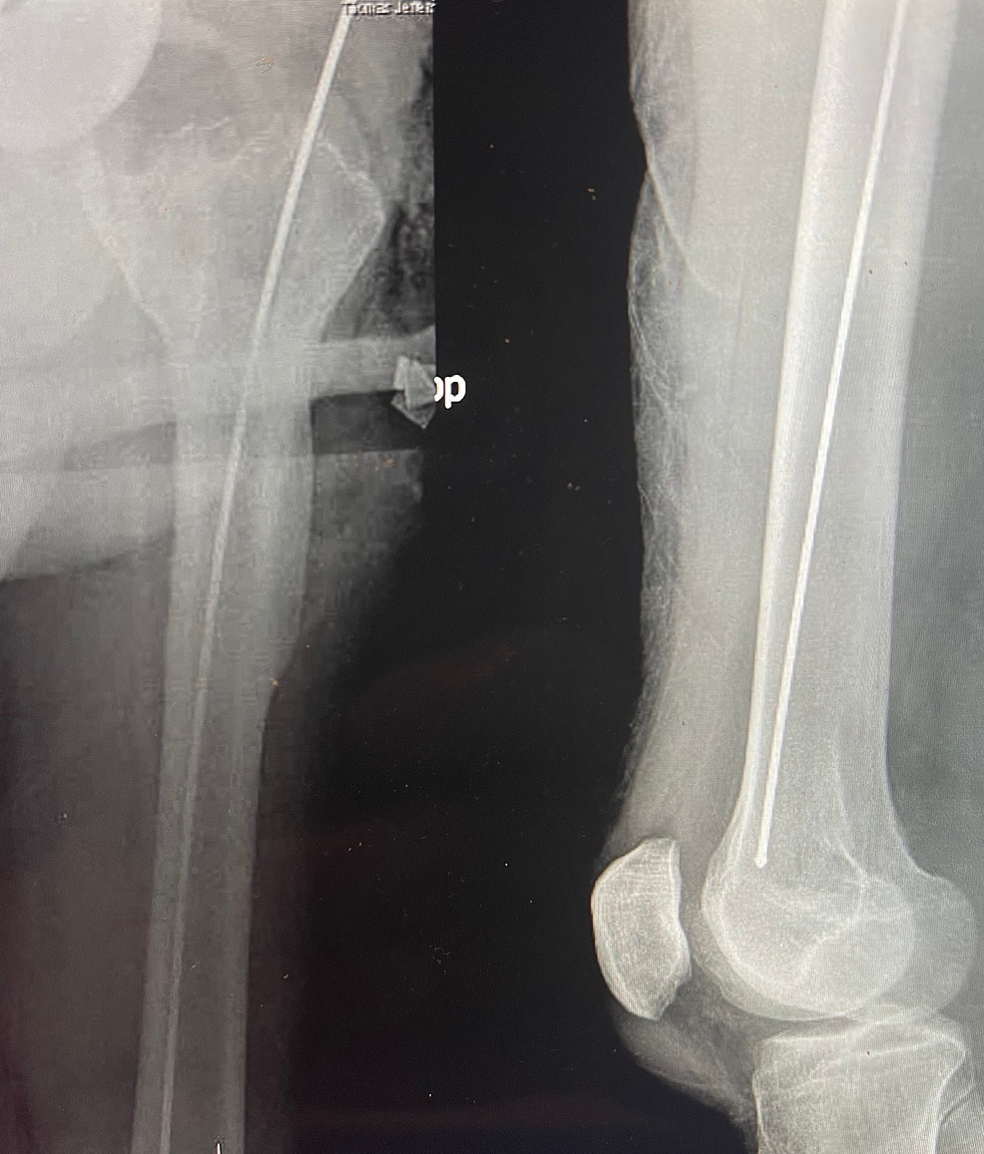
Intraoperative radiographic images demonstrating intramedullary positioning of a ball-tipped guide wire

**Figure 7:**
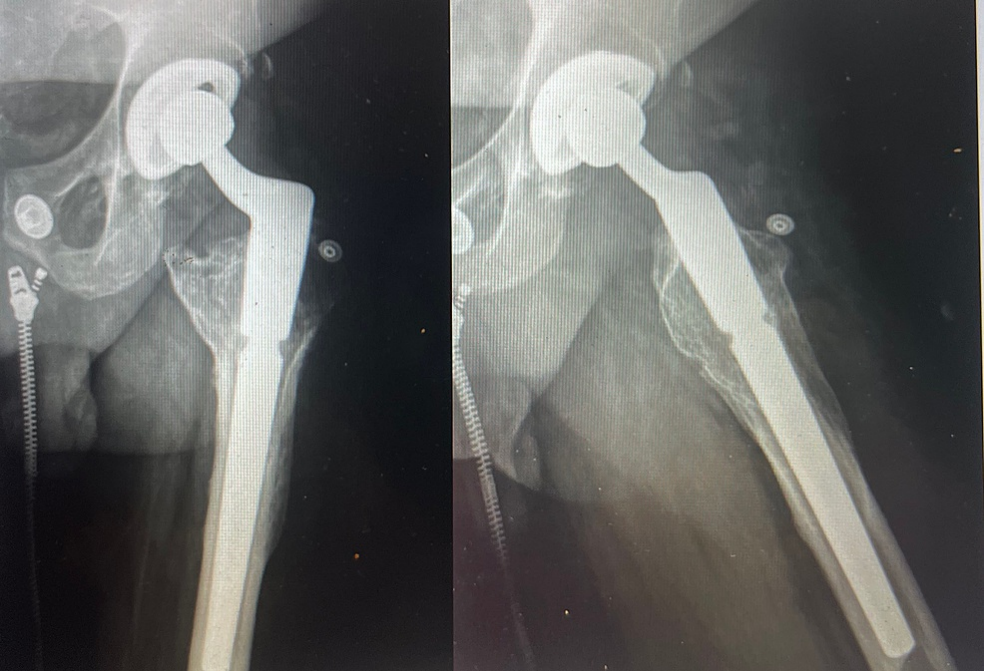
Postoperative anteroposterior and lateral radiographs demonstrating intramedullary implant positioning with improvement of hip length and offset

Next, a burr was used in order to access the true femoral canal. A ball-tipped guidewire was used with intraoperative radiographs to confirm intramedullary positioning (Figure 6). The femur was then reconstructed with a tapered, modular revision system. Care was taken to bypass the region of the perforation by greater than two cortical diameters. Postoperative radiographs (Figure 7) revealed anatomic restoration of femoral offset with 10 mm of residual leg lengthening, which was necessary to achieve prosthetic stability. The patient was instructed on protected weight-bearing for six weeks. After six months, the patient reported complete pain relief and unassisted gait with only a mild limp. The patient was last seen three years after his revision and showed an improvement in his Harris Hip Score value from 48 prior to surgery to 89 on his most recent follow-up.

## Discussion

Most of the existing literature relating to femoral perforation during total hip arthroplasty is from the 1970s and 1980s [2-3,5,7]. Talab et. al. [2] reported on 14 such cases of femoral perforation during total hip arthroplasty. Of the six patients treated with nonoperative intervention, all had at least occasional persistent pain and three relied on an assistive device for ambulation. Pellicci et. al. [3] reported on 12 cases of femoral perforation treated with nonoperative intervention. Nine of these patients were asymptomatic and without signs of mechanical failure at an average follow-up of five years. The incidence, complications, and clinical consequences of the complication with the use of modern technique and cementless components have not been well-described in the literature [1], but there has been some more recent descriptions and recommended techniques for preventing the complication as a specific risk to the direct anterior surgical approach [4,8-9]. 

We report a young patient with persistent pain and subjective weakness 12 years after femoral perforation with a cementless component. Our case demonstrates that the partial fibrous ingrowth of the component, substantial leg lengthening, loss of femoral offset, and soft tissue irritation related to the femoral perforation may lead to long-term symptoms. In our case, femoral revision resulted in correction of the position of the component, byspassing the perforation site, and correction of the leg length and femoral offset. The surgical intervention resulted in pain relief and functional improvement.

In order to avoid this devastating complication, the surgeon should strive to recognize its risk factors, execute careful preoperative planning, gain complete exposure of the proximal femur, and obtain orthogonal radiographs when concern for the complication arises [1]. When preoperative planning shows substantial femoral deformity that may predispose for the complication, the use of a relatively short femoral component may be considered. Additionally, in cases involving prior surgery with distorted proximal femoral anatomy, the use of intraoperative radiographs is advised if any doubt exists regarding the proper intramedullary positioning of the implant. 

When femoral perforation is not recognized until the postoperative period, the appropriate management is debatable. While a satisfactory outcome is possible with nonoperative intervention [3], compromised outcomes and late complications such as periprosthetic fractures are also possible [2,5-7]. Despite a 12-year implant survival, the patient described in our case lost the ability to ambulate without a cane and experienced symptoms that warranted component revision. When femoral perforation is recognized in the postoperative period after hip arthroplasty, we recommend component revision in order to avoid future complications and to optimize the patient’s outcome. It is important to note that revision of the component after the implant has achieved bony ingrowth is technically more complicated than revision of the component in the early perioperative period, prior to bony ingrowth. This report describes our technique for revision in this setting.

## Conclusions

Femoral perforation as a complication of total hip arthroplasty is a rare complication and is most often identified in the intraoperative or immediate postoperative periods. When identified at this point, the complication is corrected through component revision or repositioning with the protection of the perforation site. This case report describes a patient who presented to the office with pain and dysfunction many years after hip arthroplasty with radiographic evidence of a well-fixed component that was malpositioned and perforating the femur. The decision was made to revise the component with the goal of correction of intramedullary component positioning, restoration of femoral length and offset, pain relief, and functional improvement. Substantial pain relief and functional improvement resulted from the revision.

## References

[1] Wade FA, Parvizi J, Sharkey PF, Purtill JJ, Hozack WJ (2006). Femoral perforation complicating contemporary uncemented hip arthroplasty. J Arthroplasty.

[2] Talab YA, States JD, Evarts CM (1979). Femoral shaft perforation: a complication of total hip reconstruction. Clin Orthop Relat Res.

[3] Pellicci PM, Inglis AE, Salvati EA (1980). Perforation of the femoral shaft during total hip replacement. J Bone Joint Surg Am.

[4] Fravel W, Deskins S, Kocher T, Wood S, Bullock M (2020). A novel technique to detect femoral shaft perforation during direct anterior total hip arthroplasty. Arthroplast Today.

[5] Fredin H (1988). Late fracture of the femur following perforation during hip arthroplasty. A report of 2 cases. Acta Orthop Scand.

[6] Doyle J, Proctor P, Bessel T, Moloney MA (1989). The mechanical effects of femoral shaft perforation at total hip replacement. Int Orthop.

[7] Doyle J, Procter P, Moloney MA (1990). Femoral shaft perforation at arthroplasty: to treat or not to treat. Arch Orthop Trauma Surg.

[8] Cohen EM, Vaughn JJ, Ritterman SA, Eisenson DL, Rubin LE (2017). Intraoperative femur fracture risk during primary direct anterior approach cementless total hip arthroplasty with and without a fracture table. J Arthroplasty.

[9] Jewett BA, Collis DK (2011). High complication rate with anterior total hip arthroplasties on a fracture table. Clin Orthop Relat Res.

